# Prevalence and Associated Factors of Tinnitus: Data From the Korean National Health and Nutrition Examination Survey 2009–2011

**DOI:** 10.2188/jea.JE20140024

**Published:** 2014-09-05

**Authors:** Kyoung Ho Park, Seung Hwan Lee, Ja-Won Koo, Hun Yi Park, Kyu Yup Lee, Young Seok Choi, Kyung Won Oh, Ari Lee, Ji-Eun Yang, Sook-Young Woo, Seon Woo Kim, Yang-Sun Cho

**Affiliations:** 1 Department of Otorhinolaryngology, Catholic University of Korea, College of Medicine, Seoul, Korea; 2 Department of Otorhinolaryngology, Hanyang University, College of Medicine, Seoul, Korea; 3 Department of Otorhinolaryngology, Seoul National University Bundang Hospital, Seoul National University College of Medicine, Seongnam, Korea; 4 Department of Otorhinolaryngology-Head and Neck Surgery, Ajou University, College of Medicine, Suwon, Korea; 5 Department of Otorhinolaryngology-Head and Neck Surgery, Kyungpook National University, College of Medicine, Daegu, Korea; 6 Department of Otorhinolaryngology-Head and Neck Surgery, Chungpook University, College of Medicine, Chung-ju, Korea; 7 Division of Chronic Disease Surveillance, Korea Centers for Disease Control & Prevention, Osong, Korea; 8 Biostatistics Team, Samsung Biomedical Research Institute, Seoul, Korea; 9 Department of Otorhinolaryngology-Head and Neck Surgery, Samsung Medical Center, Sungkyunkwan University School of Medicine, Seoul, Korea

**Keywords:** tinnitus, epidemiology, associated factor, South Korea

## Abstract

**Background:**

Tinnitus is a common condition and frequently can be annoying to affected individuals. We investigated the prevalence and associated factors for tinnitus in South Korea using the data from the Korea National Health and Nutrition Examination Surveys (KNHANES) during 2009–2011.

**Methods:**

KNHANES is a cross-sectional survey of the civilian, non-institutionalized population of South Korea (*n* = 21 893). A field survey team that included an otolaryngologist moved with a mobile examination unit and performed interviews and physical examinations.

**Results:**

Among the population over 12 years of age, the prevalence of any tinnitus was 19.7% (95% CI 18.8%–20.6%). Tinnitus was more prevalent in women, and the prevalence rate increased with age (*P* < 0.001). Among those with any tinnitus, 29.3% (95% CI 27.3%–31.3%) experienced annoying tinnitus that affected daily life. Annoying tinnitus also increased with age (*P* < 0.001), but no sex difference was demonstrated (*P* = 0.25). In participants aged 40 years or older, age, quality of life, depressive mood, hearing loss, feeling of dizziness, and rhinitis were associated with any tinnitus (*P* < 0.05). Age, hearing loss, history of cardiovascular disease, and stress were associated with annoying tinnitus (*P* < 0.05).

**Conclusions:**

Tinnitus is a common condition, and a large population suffers from annoying tinnitus in South Korea. Public understanding of associated factors might contribute to better management of tinnitus.

## INTRODUCTION

Tinnitus, the perception of sound from the ears or head without an audible external source, is a symptom, rather than a disease entity, that originates internally without an external auditory input. It is relatively common in the elderly, but the actual reported prevalence varies according to the surveyed region and definition of tinnitus.^1^^,^^2^ For example, the prevalence of tinnitus was reported as 5.17% in a study from Egypt^3^ and 25.3% in a report from the 1999–2004 National Health and Nutritional Examination Survey (NHANES) in the United States (US).^4^

Nation-wide epidemiologic studies that are conducted by government organizations can provide powerful data for investigating the national prevalence of disease conditions. The Korea National Health and Nutrition Examination Survey (KNHANES) was started in 1998 to examine the general health and nutrition status of populations in South Korea. From 2009 to 2011, a total of 10 000 to 12 000 individuals were selected annually, and the participating household members were interviewed on their health and nutrition and asked to undergo a basic health assessment that included blood pressure measurements, blood and urine collection, a pulmonary function test, a dental examination, an ophthalmologic examination, and an otolaryngologic examination. In the otolaryngologic interview and examination, history of tinnitus was surveyed and otologic evaluations of the tympanic membrane (TM), hearing, and balance were conducted in participants of appropriate ages.

The present study was undertaken to determine the national prevalence of tinnitus in South Korea based on survey data obtained from the 2009 to 2011 KNHANES and to investigate associated factors. Identification and modification of these factors may aid in reducing the incidence or severity of tinnitus and, in turn, facilitate the efficient allocation of public health resources aimed at reducing the negative effects of tinnitus in everyday life.

## MATERIALS AND METHODS

### Study population and data collection

The KNHANES is an ongoing cross-sectional survey of the civilian non-institutionalized population of South Korea. A field survey team that included an otolaryngologist, an ophthalmologist, and nurse examiners for health assessment moved with a mobile examination unit and performed interviews and physical examinations. Every year, 10 000 to 12 000 individuals in about 4600 households are selected from a panel to represent the population by using a multistage clustered and stratified random sampling method that is based on the National Census Data. The participation rate of selected households in the past several cycles of KNHANES has been high, ranging from 79% to 84%. A total of 21 893 individuals, representing the 41 900 788 individuals in South Korea ≥12 years of age, participated in the survey from July 2009 to December 2011. Among them, 9653 male participants represented 20 923 340 Korean men and 12 240 female participants represented 20 977 448 women.

Written informed consent was obtained from all participants prior to the survey, and approval for this research was obtained from the Institutional Review Board of the Samsung Medical Center (IRB No. 2013-02-031).

### Survey for tinnitus and otologic examination

Participants were asked about their experiences of tinnitus. In response to the questionnaire item “Within the past year, did you ever hear a sound (buzzing, hissing, ringing, humming, roaring, machinery noise) originating in your ear?”, examiners were instructed to record ‘yes’ if a participant reported hearing an odd or unusual noise at any time in the past year. Participants who responded positively to this question were then queried concerning the resulting annoyance in their lives by the following questions: “How severe is this noise in daily life?” (not annoying/annoying (irritating)/severely annoying and causes sleep problem). The participants were grouped as having annoying tinnitus if the severity of tinnitus was annoying or severely annoying.

To determine the prevalence of TM perforation and cholesteatomatous conditions, including retraction pocket and otitis media with effusion (OME), an ear examination was then conducted with a 4-mm 0°-angled rigid endoscope attached to a CCD camera for all participants ≥4-years-of-age.

The pure tone air-conduction threshold was measured in a sound-proof booth using an automatic audiometer (GSI SA-203; Entomed Diagnostics AB, Lena Nodin, Sweden). Hearing loss was defined as over 25 decibels hearing level (dB HL) with average air-conduction hearing thresholds measured at 0.5, 1, 2, and 3 kHz according to the recommendation from the American Academy of Otolaryngology-Head and Neck Surgery.^5^

### Balance survey

Participants aged 40 years or older were asked whether they had any experience during the prior 12 months of dizziness or imbalance, subjective positional dizziness, or falls in the absence of external factors. To assess balance, participants were asked to stand on a firm surface with their feet approximately 10 cm apart for at least 15 seconds with their arms crossed, while bending their knees or moving their body to maintain balance. They were not allowed to move their feet with their eyes open (condition 1) or closed (condition 2). Next, we assessed the effect of eliminating somatosensory input on postural stability, by repeating the same procedure but on a foam pad with eyes open (condition 3) or closed (condition 4) for at least 20 seconds. The balance of an individual was scored on a pass/fail basis. A participant failed the balance test if they moved their feet, unfolded their hands, opened their eyes or required the operator to intervene to maintain their balance. As condition 4 evaluated the vestibular function to maintain balance, participants were concluded to have vestibular dysfunction if they did not pass test condition 4, even if they had passed test conditions 1–3.

### Rhinologic survey and examination

Participants were interviewed and examined for their nasal symptoms and disorders. The diagnosis of rhinitis was made when the participants had subjective symptoms of watery rhinorrhea, sneezing, itching, and nasal obstruction without a fever or a sore throat within the past year (“Within the past year, did you ever experience symptoms of rhinitis such as sneezing, runny nose, nasal obstruction, or itchy nose, without having a cold (fever or sore throat)?”). Chronic rhinosinusitis was diagnosed when the subjective symptoms of both nasal obstruction and discharge were present for more than 3 months and an intranasal endoscopic examination showed objective findings of discolored nasal drainage or polyps.^6^

### Evaluation of associated factors

As tests and questionnaires related to balance problems were performed only in participants aged 40 years or older, we analyzed the correlation between tinnitus and the associated factors in participants over that age. We initially selected 13 419 participants aged 40 years or older. Subjects with any missing data were dropped from the association analysis, leaving only 5140 complete cases.

Potential associated factors from the basic health examination and interview were evaluated for their association with the incidence and severity of tinnitus in a total of 5140 participants (2280 men and 2860 women). Data from the otolaryngologic survey and examinations, including TM perforation, hearing loss, dizziness and vestibular dysfunction, rhinitis, and chronic rhinosinusitis were evaluated. Age, sex, marital status, household income (grouped as lower, lower middle, upper middle, and upper), quality of life (evaluated by EQ-5D score from EuroQol group^7^), smoking (past/current), drinking history (more than once a month), history of stroke, experience of depressive mood, and recognition of stress (from the questionnaire) were also included. In addition, anemia (defined as hemoglobin <13 g/dL for men, and <12 g/dL for women), diabetes (defined as fasting blood glucose level >126 mg/dL), hepatitis B (measured by electrochemiluminescence immunoassay), and obesity (defined as body mass index >25 kg/m^2^) were included. Potentially associated factors were evaluated by univariable analysis. Only variables with a *P*-value ≤0.05 were selected for multivariable analysis in the logistic regression model. Associated factors for any tinnitus were evaluated in the total population and those for annoying tinnitus were evaluated among subjects with any tinnitus.

### Statistical analysis

The prevalence and 95% confidence intervals (CIs) for any and annoying tinnitus were calculated. In univariable analysis, Rao-Scott Chi-square test (using PROC SURVEYFREQ in SAS version 9.3; SAS Institute, Cary, NC, USA) and logistic regression analysis (using PROC SURVEYLOGISTIC in SAS) were used to test the association between tinnitus and risk factors in a complex sampling design. In multivariable analysis, adjusted odds ratios (ORs) with 95% CIs were calculated using logistic regression analysis (using PROC SURVEYLOGISTIC in SAS). To reflect national population estimates, sample weights were applied in all analyses. All *P*-values were two-sided, and *P* < 0.05 was considered to be statistically significant.

## RESULTS

### Prevalence of tinnitus

Among the 21 893 participants ≥12 years of age, 4523 had experienced tinnitus in the prior 12 months; the prevalence of any tinnitus was 19.7% (95% CI 18.8%–20.6%). Among 4512 participants who experienced any tinnitus, 1466 suffered from annoying tinnitus in daily life, representing 29.3% (95% CI 27.3%–31.3%) of the tinnitus population and 5.8% of the total population. Any tinnitus and annoying tinnitus increased with age (*P* < 0.001), and prevalence of any tinnitus was higher in women than men (*P* < 0.001). However, there was no sex difference (*P* = 0.25) in presence of annoying tinnitus among those with any tinnitus (Table 1).

**Table 1.** Prevalence of any and annoying tinnitus by gender and age group in participants over 12 years old (*n* = 21 893)

Characteristics	Any tinnitus				Annoying tinnitus			
	
	%^a^	Prevalence	95% CI	*P*-value	%^b^	Prevalence	95% CI	*P*-value
**Gender**								
Male	44.95	17.73	16.73–18.74	<0.001^c^	40.51	28.27	25.69–30.84	0.25^c^
Female	55.05	21.66	20.54–22.79		59.49	30.16	27.63–32.69	
**Age (years)**								
Total (≥12)	100	19.70	18.84–20.56	<0.001^d^	100	29.31	27.33–31.29	<0.001^d^
12–18	10.11	17.63	15.38–19.88		8.71	15.72	11.32–20.13	
19–29	14.40	16.67	14.82–18.51		9.15	19.49	14.59–24.40	
30–39	16.52	17.27	15.79–18.75		13.65	20.10	16.34–23.85	
40–49	16.03	16.25	14.67–17.82		13.01	24.77	20.35–29.18	
50–59	16.77	21.11	19.39–22.84		16.25	33.78	29.51–38.05	
60–69	12.57	26.43	24.54–28.32		18.79	39.26	35.07–43.45	
>70	13.60	32.12	29.94–34.30		20.43	51.65	47.68–55.62	

CI, confidence interval.

^a^Percent of Korean population over 12 years old.

^b^Percent of population with any tinnitus.

^c^Rao-Scott Chi-square test was used.

^d^For the age-related trend test, logistic regression analysis was used.

In a subset of 5140 participants ≥40 years of age in whom analysis of associated factors was performed, the prevalence of any tinnitus was 20.7% (95% CI 19.1%–22.2%). Among participants ≥40 years of age reporting any tinnitus (*n* = 1112), the prevalence of annoying tinnitus was 34.8% (95% CI 31.1%–38.5%).

### Analysis of associated factors

Experiences of any/annoying tinnitus and associated factors were investigated using univariable and multivariable analyses. In univariable analysis, any tinnitus was associated with increased stress, depressive mood, history of stroke, angina, and better quality of life. Among the otolaryngologic problems, TM perforation, hearing loss, rhinosinusitis, and balance problems (occurrence of dizziness or imbalance, subjective positional vertigo, falling attack, and vestibular dysfunction) were also associated with any tinnitus.

After multivariable-adjusted analysis, older age (OR 1.02, 95% CI 1.01–1.03), better quality of life (OR 0.99, 95% CI 0.99–1.00), hearing loss (OR 2.04, 95% CI 1.61–2.58), rhinitis (OR 1.48, 95% CI 1.20–1.84), depressive mood (OR 1.56, 95% CI 1.14–2.15), and experiencing dizziness or imbalance (OR 2.11, 95% CI 1.72–2.59) remained as associated factors of any tinnitus (Table 2). For annoying tinnitus, stress, diabetes, obesity, history of cardiac disease, hearing loss, and vestibular dysfunction were associated in univariable analysis. After multivariable-adjusted analysis, older age (OR 1.03, 95% CI 1.01–1.04), hearing loss (OR 1.55, 95% CI 1.09–2.21), history of cardiac disease (OR 5.51, 95% CI 1.04–29.20), and stress (OR 1.64, 95% CI 1.14–2.36) were associated with annoying tinnitus (Table 3).

**Table 2.** Analysis of factors potentially associated with any tinnitus in participants over 40 years old (*n* = 5140)

Variables	*n* (%^a^)	Tinnitus	Univariable analysis			Multivariable analysis^c^		
	
			*P*-value	OR	95% CI	*P*-value	OR	95% CI
**Demographic characteristics**								
Age, years (mean)^b^		57.23	<0.001	1.03	1.02–1.04	<0.001	1.02	1.01–1.03
Sex								
Male (%)	2280 (48.88)	20.33	Referent					
Female (%)	2860 (51.12)	20.99	0.62	1.04	0.89–1.22			
Marital status								
No (%)	64 (1.65)	16.27	0.42	0.74	0.36–1.53			
Yes (%)	5076 (98.35)	20.74	Referent					
Income								
Lower (%)	1208 (25.16)	20.48	Referent					
Lower middle (%)	1297 (25.65)	22.83	0.73	1.15	0.86–1.53			
Upper middle (%)	1328 (25.26)	20.70	1.00	1.01	0.76–1.36			
Upper (%)	1307 (23.94)	18.52	0.92	0.88	0.66–1.18			
**Otolaryngologic conditions (Physical examination and questionnaire)**								
Falling attack								
No (%)	5058 (98.29)	20.26	Referent			Referent		
Yes (%)	82 (1.71)	44.24	<0.001	3.13	1.75–5.58	0.20	1.42	0.83–2.43
History of dizziness								
No (%)	4169 (82.24)	17.48	Referent			Referent		
Yes (%)	971 (17.76)	35.43	<0.001	2.59	2.13–3.15	<0.001	2.11	1.72–2.59
Vertigo, subjective positional								
No (%)	5007 (97.87)	20.40	Referent			Referent		
Yes (%)	133 (2.13)	32.90	<0.01	1.91	1.26–2.92	0.15	0.73	0.47–1.13
Vestibular dysfunction								
No (%)	4978 (97.22)	20.41	Referent			Referent		
Yes (%)	162 (2.78)	29.83	0.02	1.66	1.06–2.59	0.28	0.75	0.44–1.27
Cholesteatoma								
No (%)	5021 (97.78)	20.57	Referent					
Yes (%)	119 (2.22)	25.04	0.36	1.29	0.74–2.24			
Hearing loss								
No (%)	4199 (84.80)	17.84	Referent			Referent		
Yes (%)	941 (15.20)	36.45	<0.001	2.64	2.15–3.24	<0.001	2.04	1.61–2.58
Otitis media with effusion								
No (%)	5117 (99.56)	20.59	Referent					
Yes (%)	23 (0.44)	37.08	0.08	2.27	0.91–5.67			
Rhinitis								
No (%)	4117 (79.42)	19.22	Referent			Referent		
Yes (%)	1023 (20.58)	26.26	<0.001	1.50	1.22–1.83	<0.001	1.48	1.20–1.84
Rhinosinusitis								
No (%)	4869 (94.62)	20.28	Referent			Referent		
Yes (%)	271 (5.38)	27.47	0.01	1.49	1.10–2.02	0.23	1.21	0.89–1.66
TM perforation								
No (%)	4984 (97.01)	20.13	Referent			Referent		
Yes (%)	156 (2.99)	38.07	<0.001	2.44	1.58–3.77	0.09	1.51	0.94–2.44
**Data obtained from the questionnaire**								
Alcohol drinking								
No (%)	1630 (27.60)	22.93	Referent			Referent		
Yes (%)	3510 (72.40)	19.80	0.05	0.83	0.69–1.00	0.40	1.09	0.90–1.31
Depressive mood								
No (%)	4909 (95.99)	20.17	Referent			Referent		
Yes (%)	231 (4.01)	32.57	<0.001	1.91	1.42–2.58	0.01	1.56	1.14–2.15
History of anxiety								
No (%)	5075 (98.93)	20.56	Referent					
Yes (%)	65 (1.07)	30.48	0.12	1.70	0.87–3.30			
History of cardiac disease								
No (%)	5015 (98.07)	20.48	Referent			Referent		
Yes (%)	125 (1.93)	30.28	0.02	1.69	1.07–2.66	0.31	1.27	0.80–2.01
History of stroke								
No (%)	5032 (98.46)	20.48	Referent			Referent		
Yes (%)	108 (1.54)	32.76	<0.01	1.89	1.22–2.93	0.35	1.29	0.76–2.20
Smoking, current								
No (%)	4156 (76.66)	20.92	Referent					
Yes (%)	984 (23.34)	19.84	0.54	0.94	0.76–1.16			
Smoking, past								
No (%)	3049 (55.06)	20.82	Referent					
Yes (%)	2091 (44.94)	20.48	0.81	0.98	0.83–1.16			
Stress								
No (%)	3805 (73.52)	19.55	Referent			Referent		
Yes (%)	1335 (26.48)	23.78	<0.01	1.28	1.08–1.53	0.17	1.15	0.94–1.39
Visual disturbance								
No (%)	5098 (99.38)	20.63	Referent					
Yes (%)	42 (0.62)	26.45	0.40	1.38	0.65–2.96			
Quality of life score (mean)^b^		70.05	<0.001	0.99	0.98–0.99	0.01	0.99	0.99–1.00
**Laboratory data**								
Anemia								
No (%)	4651 (91.51)	20.49	Referent					
Yes (%)	489 (8.47)	22.58	0.31	1.13	0.90–1.44			
Diabetes								
No (%)	4459 (87.31)	20.38	Referent					
Yes (%)	681 (12.69)	22.64	0.32	1.14	0.88–1.49			
Hepatitis B								
No (%)	4957 (96.31)	20.80	Referent					
Yes (%)	183 (3.69)	79.20	0.31	0.80	0.52–1.23			
Hypercholesterolemia								
No (%)	4298 (83.88)	20.36	Referent					
Yes (%)	842 (16.12)	22.25	0.28	1.12	0.91–1.37			
Hypertension								
No (%)	3204 (64.63)	19.83	Referent					
Yes (%)	1936 (35.37)	22.20	0.09	1.15	0.98–1.36			
Obesity								
No (%)	3319 (63.88)	20.93	Referent					
Yes (%)	1821 (36.12)	20.21	0.63	0.96	0.80–1.14			

CI, confidence interval; OR, odds ratio; TM, tympanic membrane.

^a^Percent of Korean population aged ≥40 years.

^b^Continuous variables were denoted by mean. In the non-tinnitus group, the mean age was 53.68, and the mean quality of life score was 74.80.

^c^Variables with *P*-value ≤0.05 in univariable analysis were selected.

Univariable and multivariable analyses were done using the logistic regression model.

**Table 3.** Analysis of potentially associated factors with ‘annoying tinnitus’ in participants over 40 years old (*n* = 1112)

Variables	%^a^	Annoying tinnitus	Univariable analysis			Multivariable analysis^c^		
	
			*P*-value	OR	95% CI	*P*-value	OR	95% CI
**Demographic characteristics**								
Age, years (mean)^b^		60.10	<0.0001	1.04	1.02–1.05	0.0007	1.03	1.01–1.04
Sex								
Male (%)	491 (48.09)	34.44	Referent					
Female (%)	621 (51.91)	35.12	0.83	1.03	0.78–1.36			
Marital status								
No (%)	13 (1.30)	45.46	0.50	1.57	0.43–5.77			
Yes (%)	1099 (98.70)	34.65	Referent					
Income								
Lower (%)	263 (24.93)	36.42	Referent					
Lower middle (%)	301 (28.33)	35.37	1.00	0.96	0.61–1.50			
Upper middle (%)	273 (25.29)	35.41	1.00	0.96	0.58–1.57			
Upper (%)	275 (21.45)	31.42	0.81	0.80	0.49–1.30			
**Otolaryngologic conditions (Physical examination and questionnaire)**								
Falling attack								
No (%)	1074 (96.34)	34.67	Referent					
Yes (%)	38 (3.66)	38.02	0.72	1.16	0.52–2.56			
History of dizziness								
No (%)	764 (69.56)	33.48	Referent					
Yes (%)	348 (30.44)	37.80	0.29	1.21	0.85–1.71			
Vertigo, subjective positional								
No (%)	1066 (96.60)	34.27	Referent					
Yes (%)	46 (3.40)	49.52	0.11	1.88	0.87–4.06			
Vestibular dysfunction								
No (%)	1064 (95.99)	34.08	Referent			Referent		
Yes (%)	48 (4.01)	51.84	0.02	2.08	1.10–3.93	0.56	1.27	0.57–2.83
Cholesteatoma								
No (%)	1080 (97.31)	34.87	Referent					
Yes (%)	32 (2.69)	32.14	0.79	0.89	0.36–2.19			
Hearing loss								
No (%)	768 (73.19)	30.25	Referent			Referent		
Yes (%)	344 (26.80)	47.18	<0.001	2.06	1.52–2.80	0.01	1.55	1.09–2.21
Otitis media with effusion								
No (%)	1100 (99.21)	34.65	Referent					
Yes (%)	12 (0.79)	53.26	0.29	2.15	0.52–8.86			
Rhinitis								
No (%)	836 (73.85)	34.52	Referent					
Yes (%)	276 (26.15)	35.56	0.80	1.05	0.73–1.51			
Rhinosinusitis								
No (%)	1037 (92.86)	34.36	Referent					
Yes (%)	75 (7.14)	40.45	0.38	1.30	0.73–2.31			
TM perforation								
No (%)	1056 (94.50)	34.07	Referent					
Yes (%)	56 (5.50)	47.19	0.08	1.73	0.94–3.17			
**Data obtained from the questionnaire**								
Alcohol drinking								
No (%)	390 (30.62)	38.53	Referent					
Yes (%)	722 (69.38)	33.15	0.11	0.79	0.59–1.05			
Depressive mood								
No (%)	1034 (93.67)	34.34	Referent					
Yes (%)	78 (6.33)	41.52	0.25	1.36	0.80–2.29			
History of anxiety								
No (%)	1091 (98.42)	34.9	Referent					
Yes (%)	21 (1.58)	30.91	0.75	0.84	0.28–2.49			
History of cardiac disease								
No (%)	1103 (99.48)	34.54	Referent			Referent		
Yes (%)	9 (0.52)	83.08	0.008	9.28	1.79–48.13	0.04	5.51	1.04–29.20
History of stroke								
No (%)	1079 (97.56)	34.51	Referent					
Yes (%)	33 (2.44)	46.04	0.28	1.62	0.68–3.88			
Smoking, current								
No (%)	919 (77.59)	35.19	Referent					
Yes (%)	193 (22.41)	33.43	0.69	0.93	0.63–1.36			
Smoking, past								
No (%)	661 (55.46)	34.28	Referent					
Yes (%)	451 (44.54)	35.44	0.74	1.05	0.78–1.43			
Stress								
No (%)	772 (69.54)	31.95	Referent			Referent		
Yes (%)	340 (30.46)	41.29	0.02	1.50	1.07–2.09	<0.01	1.64	1.14–2.36
Visual disturbance								
No (%)	1100 (99.21)	34.71	Referent					
Yes (%)	12 (0.79)	44.76	0.51	1.52	0.43–5.40			
Quality of life score (mean)^b^		68.16	0.06	0.99	0.98–1.00			
**Laboratory data**								
Anemia								
No (%)	997 (90.73)	34.85	Referent					
Yes (%)	115 (9.27)	34.20	0.90	0.97	0.62–1.53			
Diabetes								
No (%)	954 (86.10)	33.43	Referent			Referent		
Yes (%)	158 (13.90)	43.22	0.03	1.52	1.03–2.24	0.26	1.25	0.85–1.84
Hepatitis B								
No (%)	1077 (96.91)	35.01	Referent					
Yes (%)	35 (3.09)	27.91	0.44	0.72	0.31–1.65			
Hypercholesterolemia								
No (%)	924 (82.65)	33.58	Referent					
Yes (%)	188 (17.35)	40.57	0.16	1.35	0.89–2.05			
Hypertension								
No (%)	660 (62.02)	32.58	Referent					
Yes (%)	452 (37.98)	38.41	0.07	1.29	0.98–1.70			
Obesity								
No (%)	719 (64.68)	37.11	Referent			Referent		
Yes (%)	393 (35.32)	30.56	0.05	0.75	0.56–1.00	0.06	0.75	0.56–1.01

CI, confidence interval; OR, odds ratio; TM, tympanic membrane.

^a^Percent of Korean population aged ≥40 years.

^b^Continuous variables were denoted by mean. In the non-annoying tinnitus group, the mean age was 55.70, and the mean of quality of life score was 71.06.

^c^Variables with *P*-value ≤0.05 in univariable analysis were selected.

Univariable and multivariable analyses were done using the logistic regression model.

## DISCUSSION

This is the first epidemiologic study to investigate the prevalence and associated factors of tinnitus based on representative data from a government-centered survey in South Korea. Accurate epidemiologic information may contribute to the proper provision of health care, preventive screenings, and rehabilitative services.

Tinnitus is the perception of sound from the ears or head without an audible external source and is known to be more common among older adults.^8^ The severity of tinnitus ranges from barely noticeable (any tinnitus) to debilitating (annoying tinnitus). Patients who experience tinnitus often report significant associated morbidities such as lifestyle detriment, emotional difficulties, sleep deprivation, work hindrance, and interference with social interaction.^4^

Several population-based studies have attempted to estimate the prevalence of tinnitus, and the reported prevalence from each region varies partly due to the different characteristics of the surveyed population or definition of tinnitus, such as duration, frequency, and severity. In a questionnaire-based survey conducted in England in 1978, the prevalence of subjective tinnitus was 15.5%–18.6%, and the prevalence of annoying tinnitus was 0.4%–2.8% in respondents over 17 years of age.^9^ A 1989 study from Sweden and a 1996 study from Italy reported the prevalence of subjective tinnitus as 14.2% and 14.5%, respectively.^10^^,^^11^ Data from the National Health Interview Survey in 1996 revealed that tinnitus was experienced by 35–50 million adults in the US, and 2–3 million reported symptoms that were severely debilitating.^12^ The overall prevalence of tinnitus in Egypt was 5.17%, and the highest age-specific prevalence was 17.7%, among subjects above 60 years of age.^3^ With regard to Asian countries, epidemiologic studies from Japan^1^ in a population aged 65 years or older and from China^13^ in subjects over 10 years of age reported that the overall prevalence of tinnitus was 18.6% and 14.5%, respectively. In the 2004 NHANES, a cross-sectional tinnitus study of 14 178 participants in the US, the prevalence of experiencing any tinnitus and frequent tinnitus was 25.3% and 7.9%, respectively. The prevalence of any and frequent tinnitus increased with age, peaking at 31.4% and 14.3%, respectively, at 60–69 years of age (Table 4).^4^ In the present study, the prevalence of any tinnitus was 19.7%, and that of annoying tinnitus was 5.8% in those over 12 years of age.

**Table 4.** Prevalence of tinnitus in previous reports

Authors	Year	Sample	Prevalence of any tinnitus	Prevalence of severe tinnitus
MRCIHR^9^	1981	England, 19 000 persons aged over 17 years	17.0%	8.0%
Axelsson et al^10^	1989	Sweden, 2378 persons aged 20–80 years	14.2%	2.4%
Quaranta et al^11^	1996	Italy, 2170 persons aged over 18 years	14.5%	
Shargorodsky et al^4^	2010	USA, 1999–2004 NHANES, 14 178 persons aged over 20 years	25.3%	7.9%
Michikawa et al^1^	2010	Japan, 1320 persons aged over 65 years	18.6%	
Kehdr et al^3^	2010	Egypt, 8484 persons aged over 6 years	5.17%	0.8%
Xu et al^13^	2011	China, 6333 persons aged over 10 years	14.5%	
Fuji et al^32^	2011	Japan, 14 423 persons aged 45–79 years	11.9%	0.4%
This study		South Korea, 21 893 persons aged over 12 years	19.7%	5.8%

MRCIHR, Medical Research Council’s Institute of Hearing Research; NHANES, National Health and Nutrition Examination Survey.

In a US population-based longitudinal study conducted in Beaver Dam, Wisconsin, the 5-year incidence of tinnitus among those free of tinnitus at baseline was 5.7%, and the 10-year cumulative incidence of tinnitus was 12.7%, in 2922 participants aged 48 to 92 years. Risk factors for occurrence of tinnitus included hearing loss, high serum total cholesterol, history of head injury, otosclerosis, arthritis, and history of smoking.^2^^,^^8^

A clear relationship has been established between hearing loss and tinnitus, and the majority of tinnitus patients have some degree of hearing loss. Since hearing loss and tinnitus are so closely related, populations with more prevalent hearing loss have a corresponding greater prevalence of tinnitus.^14^ In a report from Egypt in 2010, 53% of tinnitus patients reported hearing loss.^3^ In a report from China,^13^ the prevalence of tinnitus was 30.0% and 6.0%, respectively, among people with and without hearing impairment. In the latter study, there was a significant relationship between high-frequency hearing impairment (>25 dB HL at 4 kHz) and high-pitched tinnitus (OR 6.61). History of middle ear infections and noise exposure were also risk factors (OR 5.90 and 2.74, respectively). In the 1999–2004 NHANES, frequent tinnitus was also associated with low-mid-frequency (OR 2.37, 95% CI 1.76–3.21) and high-frequency (OR 3.00, 95% CI 1.78–5.04) hearing impairment.^4^ Kim et al reported that, even if patients with tinnitus do not have any subjective hearing impairment, most of them have high-frequency hearing loss (above 2 kHz) and/or extended-high-frequency hearing loss (above 8 kHz).^15^ Our results also confirm that unilateral or bilateral hearing loss (>25 dB HL) was associated with any tinnitus (OR 2.04) and annoying tinnitus (OR 1.55).

Regarding the relationship between tinnitus and vestibular dysfunction, our results indicate that any tinnitus, but not annoying tinnitus, was associated with experience of dizziness or imbalance (OR 2.11, 95% CI 1.72–2.59). The Blue Mountains Hearing Study also reported dizziness as a prognostic factor for incident tinnitus.^16^ Although the mechanism behind this association is still unclear, a possible explanation is that the dysfunction of the vestibular labyrinth might have an association with the auditory pathways that causes tinnitus.

The prevalence of hearing loss and tinnitus increases with age. About 27% of all individuals between the ages of 60 and 69 years and 45% of those persons 70 years and older have subjective hearing loss in South Korea.^17^ The 1999–2004 NHANES data have established that the odds of any tinnitus (OR 1.67, 95% CI 1.44–1.94) and frequent tinnitus (OR 5.43, 95% CI 3.93–7.50) appear to peak at 60–69 years of age compared with subject under 50-years-of-age.^4^ Although hearing loss is commonly associated with tinnitus in older patients, other medical conditions become increasingly prevalent in this population and must be considered as potential causes of tinnitus. These conditions include vascular disease, middle-ear disease, diabetes, hypertension, autoimmune disorders, and degenerative neural disorders with or without concomitant hearing loss.^14^^,^^18^^,^^19^

Differences in the prevalence of tinnitus according to gender have been amply demonstrated. The US National Center for Health Statistics in 1996 reported that prevalence of tinnitus was 12% for men and 7% for women over 65 years of age.^12^ The 1999–2004 NHANES demonstrated that prevalence of any tinnitus for men and women was 26.1% and 24.6%, respectively, in participants over the age of 20 years, and that this difference was significant.^4^ In our study, prevalence of any tinnitus was 17.7% for men and 21.7% for women (*P* < 0.001) in participants over the age of 12 years, and this tendency was consistent through the surveyed period. Similarly, the prevalence of annoying tinnitus was 28.3% for men and 30.2% for women among those with any tinnitus (*P* = 0.25). These results might be attributed to a more stressful cultural situation for women in South Korea, which demands obedience and more roles in the family. A survey from Japan also demonstrated that tinnitus was more prevalent in women than in men, though without statistical significance.^1^

The neurophysiologic model of tinnitus postulates that tinnitus emerges as a result of the interaction of a number of neural subsystems. The auditory pathways plays a role in the perception of tinnitus, whereas the limbic system is responsible for the development of tinnitus annoyance. Therefore, psychological stress, anxiety, and depressive mood can be associated with the development and severity of tinnitus.^20^^–^^23^ Epidemiologic studies have demonstrated that stress, anxiety, depressive mood, and sleep deprivation were associated with tinnitus,^24^ and 36%–62% of tinnitus patients were found to be depressed in previous studies.^25^^,^^26^

In the 2011 Beaver Dam offspring study, depressive symptoms (OR 2.04, 95% CI 1.50–2.76) and the use of anti-depressant medications (OR 1.58, 95% CI 1.17–2.14) were significantly associated with tinnitus.^27^ The 1999–2004 NHANES also demonstrated the association of generalized anxiety and major depressive disorders with tinnitus among the 2265 participants between the ages of 20 and 39 years of age. After multivariable adjustment, participants with generalized anxiety disorder had higher multivariate-adjusted odds of any tinnitus (OR 2.66, 95% CI 1.32–5.34) and frequent tinnitus (OR 6.07, 95% CI 2.33–15.78) than those without anxiety disorder. As for depressive disorder, only the odds of any tinnitus remained significant after adjustment (OR 2.01, 95% CI 1.24–3.25).^4^ Epidemiologic studies from Japan in a population over 65 years of age^1^ and from Egypt in the general population over 6 years of age^3^ also showed that depressive mood and emotional stress were associated with tinnitus. Our results also demonstrated that occurrence of any tinnitus is associated with depressive mood (OR 1.56, 95% CI 1.14–2.15), and annoying tinnitus with stress (OR 1.64, 95% CI 1.14–2.36).

Tinnitus can result in sleep deprivation, decreased work productivity, and overall lifestyle detriment,^26^^,^^28^^,^^29^ which consequently leads to psychological distress and depression.^30^ Likewise, major depressive and generalized anxiety disorders may exacerbate tinnitus, while their treatment might alleviate tinnitus.^31^ Depression and anxiety scores have been reported to be higher at the early stage of tinnitus development.^20^

In our survey, history of cardiac disease has a strong association with annoying tinnitus (OR 5.51, 95% CI 1.04–29.20). This association has been reported in several studies,^1^^,^^11^^,^^32^ but the mechanism is not clear. The vascular dysregulation hypothesis is one possible theory for the development of tinnitus.^33^ Dysregulation is often associated with a reduced cardiac output, as well as with reflex activation of vasoconstrictive systems, including the sympathetic nervous system and renin-angiotensin-aldosterone system.^33^ The results of the present study support the hypothesis that cardiovascular disorders may be associated with severe tinnitus.

As this study utilized cross-sectional data from annual surveys, we were unable to obtain incidence estimates and risk factors. Instead, we were able present only prevalence rates and associated factors of tinnitus, which is a limitation of this study. Another limitation of the study is that relatively few participants were included in the association analysis. A large number of cases were dropped due to missing data and age restriction of the vestibular survey, which only included participants with complete data aged 40 years or older. Among a total of 13 419 participants aged 40 years or older, 5140 had a complete data set. As participants in the association subset were not significantly different from the total participants in the prevalence of any/annoying tinnitus and male to female ratio, important characteristics of the population who were enrolled in the association analysis were not largely changed by exclusion of the missing data.

In summary, the overall prevalence of any tinnitus is 19.7%, and the prevalence of annoying tinnitus is 5.8% in the general population of South Korea. Tinnitus is more prevalent in women than in men, and the prevalence increases with age. Analysis of associated factors demonstrated that any tinnitus is associated with better quality of life, hearing loss, rhinitis, depressive mood, and feelings of dizziness or imbalance. Annoying tinnitus is associated with hearing loss, history of cardiac disease, and stress.

This study demonstrates that tinnitus is a common condition and that a large population suffers from annoying tinnitus in South Korea. Public acknowledgement and further intervention to modify factors associated with tinnitus are required for prevention and management of the condition, for which no known cure exists.
